# Soil carbon (C), nitrogen (N) and phosphorus (P) stoichiometry drives phosphorus lability in paddy soil under long-term fertilization: A fractionation and path analysis study

**DOI:** 10.1371/journal.pone.0218195

**Published:** 2019-06-24

**Authors:** Muhammad Qaswar, Waqas Ahmed, Huang Jing, Fan Hongzhu, Shi Xiaojun, Jiang Xianjun, Liu Kailou, Xu Yongmei, He Zhongqun, Waleed Asghar, Asad Shah, Huimin Zhang

**Affiliations:** 1 National Engineering Laboratory for Improving Quality of Arable Land, Institute of Agricultural Resources and Regional Planning, Chinese Academy of Agricultural Sciences, Beijing, China; 2 National Observation Station of Qiyang Agri-ecology System, Institute of Agricultural Resources and Regional Planning, Chinese Academy of Agricultural Sciences, Qiyang, Hunan, China; 3 Soil and Fertilizer Research Institute, Sichuan Academy of Agricultural Sciences, Chengdu, China; 4 College of Resources and Environment, Southwest University, Chongqing, China; 5 Jiangxi Institute of Red Soil, National Engineering and Technology Research Center for Red Soil Improvement, Nanchang, China; 6 Institute of Soil, Fertilizer and Agricultural Water Conservation, Xinjiang Academy of Agricultural Sciences, Urumqi, China; 7 College of Horticulture, Sichuan Agricultural University, Chengdu, China; 8 School of Environment, Beijing Normal University, Beijing, China; University of Minnesota, UNITED STATES

## Abstract

Soil C:N:P stoichiometry plays a vital role in nutrient cycling in ecosystems, but its importance to P transformation in paddy soil remains unclear. We investigated the effect of soil C:N:P stoichiometry on P mobility and uptake under long-term fertilization. Three treatments, CK (no fertilization), NPK (inorganic nitrogen, phosphorus and potassium fertilization) and NPKM (combined inorganic NPK fertilizer and manure application), were selected from two long-term experiments of paddy soil that were initiated in 1991 and 1982 in Chongqing and Suining, respectively. The results showed that in comparison the control treatment, under long-term fertilization, soil pH decreased. In comparison with the NPK and CK treatments, the NPKM treatment significantly increased soil nutrient contents, P uptake and phosphatase activities. In comparison to the CK treatment, the NPK and NPKM treatments significantly decreased soil C:N, C:P and N:P ratios. In comparison to NPK and CK treatments, the NPKM treatment decreased residual-P at both sites. Compared with CK treatment, the NPKM treatments increased labile-P and moderately labile-P by 987% and 144%, respectively, and NPK treatment increased these factors by 823% and 125%, respectively, at the Chongqing site. At the Suining site, with NPKM treatment, increases in labile-P and moderately labile-P were 706% and 73%, respectively, and with NPK treatment, the increases were 529% and 47%, respectively. In contrast, non-labile-P was significantly decreased with NPKM treatment in comparison to that with NPK and CK treatments. Moreover, increases in soil C:N and C:P ratios decreased the labile-P pools and increased non-labile-P pools. A path analysis indicated that soil C:N:P stoichiometry indirectly controlled P uptake by directly affecting P transformation from non-labile to labile-P pools. Moreover, the non-labile-P in soil with high SOM and P content directly affected P uptake, indicating that soil P transformation is mainly driven by soil C and P in paddy soil. In conclusion, understanding mechanism of P mobility influenced by soil C:N:P stoichiometry could be helpful to manage soil P fertility under long-term fertilization in paddy soils of these regions.

## Introduction

Crop productivity is directly affected by P availability in agricultural soils [1]. However, in some agricultural soils, the concentration of total P is naturally high, which can limit plant growth due to the low solubility and rapid conversion of P compounds to unavailable or poorly available P after fertilizer application [2]. A synthetic chemical fertilizer application can ensure sufficient availability of P for plant uptake [3]. However, there has been growing confirmation that long-term excessive application of inorganic P fertilizers decreases P use efficiency that may result in excessive accumulation of P in soil [4].Specifically, in intensive cultivation areas, during surface runoff or erosion the P-rich soil particles may reach surface water and cause eutrophication [5,6].

The excessive use of inorganic fertilizers could be reduced by animal and green manure application in fields [7,8]. Organic amendments can increase the P recovery in soil by decreasing the formation of irreversible adsorption complexes in soil [9,10] or by increasing the P mobility in soil [11]. Additionally, organic amendments not only provide macronutrients and micronutrients, but also improve soil physical and chemical properties by increasing SOM [12]. However, in practice the rate of manure addition to fields is mostly calculated based on N input rates, which may cause excessive P addition with manure, especially under long-term fertilization [13]. Therefore, the combined application of inorganic fertilizers and manure could be beneficial to increasing the efficiency of applied P and thus improving crop productivity. The arable land in the Chongqing and the Suining (southern China) are mainly paddy soils, which are mostly cultivated under rice based cropping systems [14,15]. Paddy soils, especially in the southern regions of China, are inherently low in available P due to acidification. Therefore, to meet plant P requirements, excessive inorganic fertilization is a common practice in these regions, which causes environmental P losses and degraded soil fertility [16]. In these regions, previous studies mainly focused on long-term fertilization effect on crop yields and soil fertility [14,17], and recommended, integrated nutrient management through the combination of applying inorganic fertilizer and manure addition to improve soil P fertility [18]. Most existing studies have clearly shown that the combined application of inorganic and manure increases the availability of P and crop yield [19], but the mechanism that causes the redistribution of residual-P under long-term fertilization in paddy soils is not well studied [20]. There could be many factors regulating P in paddy soil under long-term fertilization. In a previous study, Ahmed et al. [21] found that the combined application of manure and inorganic fertilizers increased the labile-P fraction, and different soil properties (pH, SOM, total P and Olsen-P) showed different relationships with P fractions, however, the authors did not explain the mechanism of P pools transformation, mobility and uptake in paddy soils associated with soil nutrient stoichiometry. One of the most important factors for P mobility in paddy soil could be soil nutrient stoichiometry, coupling and interactions of the C, N and P cycles [22–24]. In recent years, great progress related to C:N:P stoichiometry in terrestrial ecosystems with focuses on microorganisms, plant leaves and litter, has been made [25,26]. Therefore, C:N:P stoichiometry could be used as a powerful tool to understand nutrient cycling and processes in soil [27]. Tian et al. [28] also reported that soil C:N:P stoichiometry could be a potential indicator for assessing soil nutrient status during soil development. However, the impact of soil C:N:P stoichiometry on P cycling, especially in paddy soil, is poorly understood.

The phosphorus that is applied to soil goes through many complex transformations (desorption-adsorption and dissolution-precipitation) controlled by soil properties such as pH, SOM and exchangeable iron-aluminum (Fe-Al) hydroxides [29,30]. The sequential procedure of P extraction developed by Hedley et al. [31], extracts the different fractions of P that is bound with complex organic and inorganic compounds using solutions of different strengths for the P extraction [32]. The Hedley method of P fractionation is one of the comprehensive methods that is used to investigate the dynamics of P lability and the P cycle in soil [33]. However, the sequential procedure of P fractionation alone does not provide insight into the transformation of P pools affected by different treatments and soil factors [34]. Path analysis could be used as a valuable tool to investigate the interrelationship between soil P pools in different soils and management practices [35]. Therefore, the aim of this study was to investigate the influence of soil nutrient stoichiometry on P pool transformation and P mobility in paddy soil after long-term fertilization using a structural equation modeling (SEM) pathway technique.

## Materials and methods

### Experimental design and site description

In this study, we selected two sites from long-term experiments located in Chongqing (106°24’33”E, 29°48’36”N) and Suining (105°03’26”E, 30°10’50”N) that were initiated in 1991 and 1981, respectively. These experimental sites belong to national public welfare research institutions. Research at these experiment sites was carried out with the permission of the Institute of Agricultural Resources and Agricultural Regional Planning, Chinese Academy of Agricultural Sciences, as cooperative research agreements were signed with these experimental sites. Climate at the Chongqing site is subtropical, and the climate at the Suining site is subtropical humid. The initial soil physical and chemical properties and climatic conditions of the experimental sites are given in Table 1. Soils in both sites are Purple-Udic Cambisols according to Chinese soil classification [36]. The cropping systems at both sites were rice-wheat double cropping systems. Hybrid indica rice was planted in the rice season at both sites, and we used data from only the rice season. Treatments at both sites were arranged in a randomized complete block design (RCBD) with three replicates. Each plot was separated by permanent cemented ridges. In this study, the three selected treatments were common at both sites. The treatments included no fertilization (CK), inorganic fertilization (NPK) and inorganic fertilizer plus manure (NPKM). The rates of fertilization are shown in Table 2. The chemical fertilizers applied at both sites were urea for N, calcium superphosphate for P and potassium sulfate for K. In the NPKM treatment, a half dose of the fertilizers was applied from an inorganic fertilizes, and the remaining half dose of nutrients was supplemented with pig fecal urine at the Chongqing site and fresh pig manure at the Suining site based on N content. The remaining P and K application rates were adjusted by inorganic fertilizers. In both experiments, phosphorus fertilizer and manure were applied as a basal fertilization before transplantation of the seedlings, and half of the N and K fertilizers were applied as a basal application. The remaining half were dressed in two splits at the tillering and panicle initiation stages of the crop. The nutrient content of the pig manure was 0.2% N, 0.17% P_2_O_5_, and 0.1% K_2_O, and the nutrient content of the pig fecal urine was 1.1% N, 0.9% P_2_O_5_, and 0.4% K_2_O experiment. The rice seedlings were transplanted in the middle of May, and the crop was harvested at the end of August at full maturity.

**Table 1:** Experiment initiation date, climate and initial soil properties at the two long-term experimental sites.

Parameters	Chongqing site	Suining site
Initiation year	1991	1982
Latitude (N)	29°48′36″	30°10′50″
Longitude (E)	106°24′33″	105°03′26″
Climate	Subtropical	Subtropical humid
Mean annual temperature (°C)	18.3	18.5
Mean annual precipitation (mm)	1106	927
Cropping system	Rice-wheat	Rice-wheat
Soil texture	Sandy loam	Clay loam
Soil pH	7.7	8.0
SOM (g kg^-1^)	24.2	15.9
TN (g kg^-1^)	1.25	1.1
AN (mg kg^-1^)	93	66.3
TP (g kg^-1^)	0.67	0.4
AP (mg kg^-1^)	4.3	3.9
TK (g kg^-1^)	21.1	26.9
AK (mg kg^-1^)	88	130

Abbreviations: SOM soil organic matter; TN total nitrogen; AN available N; TP total phosphorus; AP available phosphorus; TK total potassium; AK available potassium

**Table 2:** Annual fertilizer input rates (kg ha^−1^) for the crop at the long-term experimental sites of two typical croplands in China.

Sites	Fertilizer application (N-P_2_O_5_-K_2_O)		
	CK^1^	NPK^2^	NPKM^3^*
Chongqing	0-0-0	150-60-60	150-60-60
Suining	0-0-0	120-60-60	120-60-60

^1^ CK: unfertilized (control)

^2^ NPK: inorganic nitrogen, phosphorous and potassium

^3^ NPKM: inorganic NPK plus manure.

* Roasted chicken manure applied at the rate of 1500 kg ha^−1^ at the Chongqing site, fresh pig manure applied at the rate of 15000 kg ha^-1^ at Suining site.

### Sampling and chemical analysis

After crop harvest, plant samples were air dried, and the yield was measured and the total P content in the plant samples was measured after oven drying at 60°C to calculate the P uptake. Soil samples were collected in the first week after the rice harvest in 2017. Composite soil samples were collected at a 0–20 cm soil depth from four randomly selected locations in each replicate block of treatments. Subsamples from each composite sample were stored at 4°C to prevent the moisture losses and to determine phosphatase activities within a week of sampling. Apart from the composite samples, the other samples were air dried and sieved < 2 mm to determine the soil chemical properties. Soil pH was determined in suspensions of water: soil (2.5: 1) [37]. Conventional routine methods [38] were used to analyze the soil organic carbon (SOC), total nitrogen (TN) and total phosphorus (TP). The wet oxidation method was used to determine SOC [39], and the Kjeldahl digestion-distillation method was used to determine soil total N. Soil total P was measured by the molybdenum blue colorimetric method using a HClO_4_-H_2_SO_4_ solution for digestion. Mineral N, also known as alkali hydrolysable N, was determined according to Lu [37], and available P was measured according to Olsen et al. [40]. Except for the refrigerated sample, the soil moisture contents of the samples were measured by oven drying at 105°C for 24 h before processing the soil samples for enzyme activities. The activities of acid phosphomonoesterase (AcP) and phosphodiesterase (PD) were determined according to Tabatabai [41]. The substrate solution used for AcP was *p*-nitrophenyl phosphate and the pH of the buffer solution was 6.5. The substrate solution used for PD was bis-p-nitrophenyl phosphate, and the pH of the buffer solution was 8. The activities of Acp and PD were expressed as mg p-nitrophenol kg^−1^ soil h^−1^.

### Phosphorus fractionation

Organic and inorganic fractions of soil phosphorus were determined using the Hedley et al. [31] fractionation method modified by Tiessen and Moir [42]. Briefly, the soil samples were divided into three replicates each of 1 g soil and sequential extraction procedure was carried out as follows: NaHCO_3_-P was extracted by continuous shaking for 16 h after adding 0.5M NaHCO_3_ at pH 8.5 in 1 g soil. Then, shaking continued with 0.1M NaOH for 16 h to extract NaOH-P in solution. Next, shaking was continued with 1 M HCL for 16 consecutive hours to obtain diluted HCl-P and then heated with 10 ml of concentrated HCl at 80°C in a water bath for 10 minutes after adding 5 ml 12M HCl, a final volume of 50 ml was made with deionized water to obtain concentrated HCl-P (conc. HCl-P). Finally, to obtain residual-P, the residual soil samples were mineralized by adding 300 μl of concentrated H_2_SO_4_ in each soil sample of 30 mg followed by heating at 350°C for 3 h. The tubes were centrifuged at 25,000×g and 4°C for 10 minutes between two consecutive steps. The supernatant was passed through 0.45 μm cellulose nitrate filters, and extra soil particles were recovered by washing filters with extracted solution used in the following steps. Both organic and inorganic P (by taking the difference between total P after digestion with persulfate and inorganic P by colorimetry) levels were determined by measuring with 0.5 M NaHCO_3_, 0.1 NaOH and 12 M HCl extracts.

### Statistical analysis

The soil C:N, C:P and N:P ratios were calculated from SOC, total N and total P. We used one-way ANOVA followed by Tukey’s HSD test at the *P* = 0.05 level for each variable to evaluate the significant differences among the different treatment means [43]. The main effects of the fertilizer treatments, sites and their interactions were evaluated by two-way ANOVA. To determine the relationship between soil C:N:P stoichiometry and the P pools, linear regression analysis was performed using SPSS 16.0 software (SPSS, Chicago, IL, USA). Redundancy analysis (RDA) and variance partitioning analysis were performed using Canoco (version 5.0) software. Structure equation modeling (SEM) is a statistical procedure to determine the direct and indirect effects and estimates the partial contribution of explanatory variables [44,45]. We used SEM to explore the hypothesized linkage among C:N:P stoichiometry, P pools and P uptake using the AMOS package with SPSS 16.0 software (SPSS, Chicago, IL, USA).

## Results

### Effect of long-term fertilization on soil properties and soil C:N:P stoichiometry

**Fig 1:**
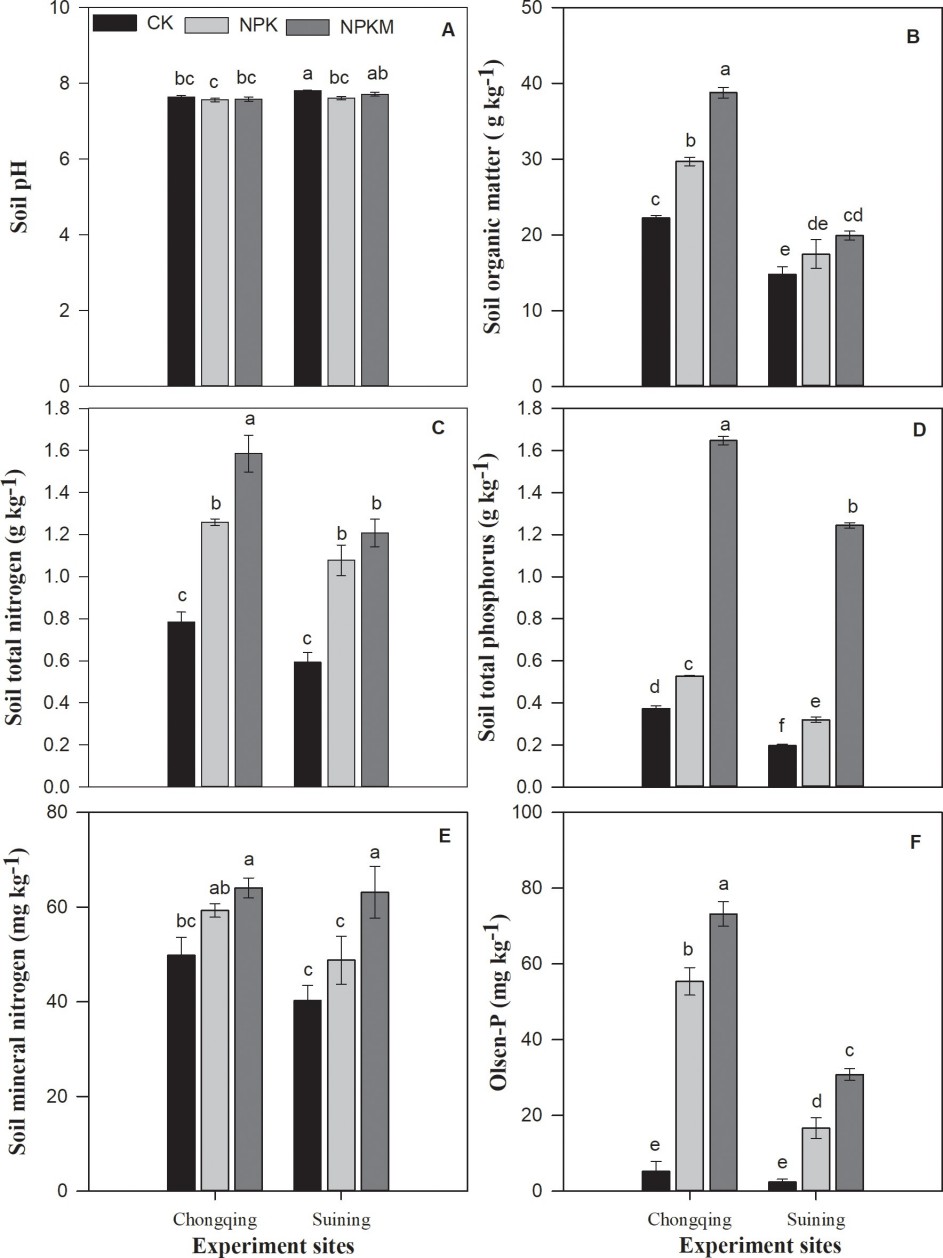
Effect of long-term fertilization on soil pH (A), organic matter (B), total nitrogen (C), total phosphorus (D), mineral nitrogen (E) and Olsen-P (F) in paddy soils. Error bars represent ± standard deviations; different lower-case letters over the bars indicate significant (*P* ≤ 0.05) differences according to Tukey's HSD test.

The interaction of soil × fertilization significantly *(P* ≤ 0.05) affected soil properties and soil C:N:P stoichiometry except soil pH and mineral N. However, the main factors (site and fertilizer treatments) significantly *(P* ≤ 0.05) influenced pH and mineral N. Among both soils, soil pH ranged from 7.8 to 7.6 (Fig 1A). At the Suining, the pH in the control (CK) was significantly higher than the pH in the fertilizer treatments (NPK and NPKM). At both sites, in comparison to the CK and NPK treatments, the combined application of inorganic fertilizer plus manure (NPKM) significantly *(P* ≤ 0.05) increased soil organic matter (SOM), total N, total P, mineral N and Olsen-P. Soil total P at the NPKM treatment was more than double that in NPK and CK treatments at both sites. Compared with the CK treatment, in the NPKM treatment increased SOM, total N, total P, mineral N and Olsen-P by 74.4, 102, 343, 29 and 1307% at the Chongqing site and by 34.4, 104, 532, 57 and 1194% respectively at the Suining. Compared with the CK treatment, the NPKM treatment increased SOM, total N, total P, mineral N and Olsen-P by 34, 60.4, 42, 19, 965% respectively, at the Chongqing site and by 18, 82, 62, 21 and 597% respectively at the Suining site.

**Fig 2:**
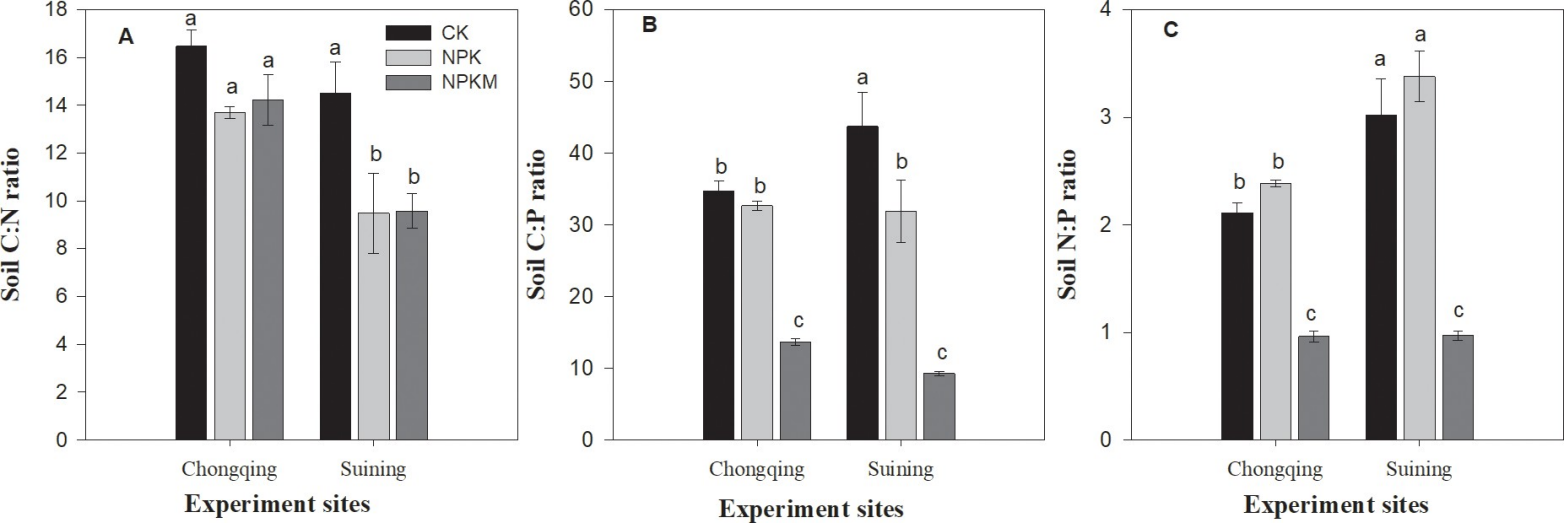
Effect of long-term fertilization on soil C:N (A), C:P (B) and N:P (C) ratios in paddy soils. Error bars represent ± standard deviations; different lower-case letters over the bars indicate significant (*P* ≤ 0.05) differences according to Tukey's HSD test.

Changes in soil total nutrient contents under long-term fertilization changed soil C:N:P stoichiometry in both soils. In comparison to no fertilizer and the inorganic fertilizer application, the long-term combined application of manure and inorganic fertilizer decreased the soil C:N, C:P and N:P ratios (Fig 2). However, the highest C:N and C:P ratio was in the CK treatment, and the highest N:P ratio was in NPK treatment at both sites.

### Effect of long-term fertilization on phosphatase activities, phosphorus fractions and uptake

**Fig 3:**
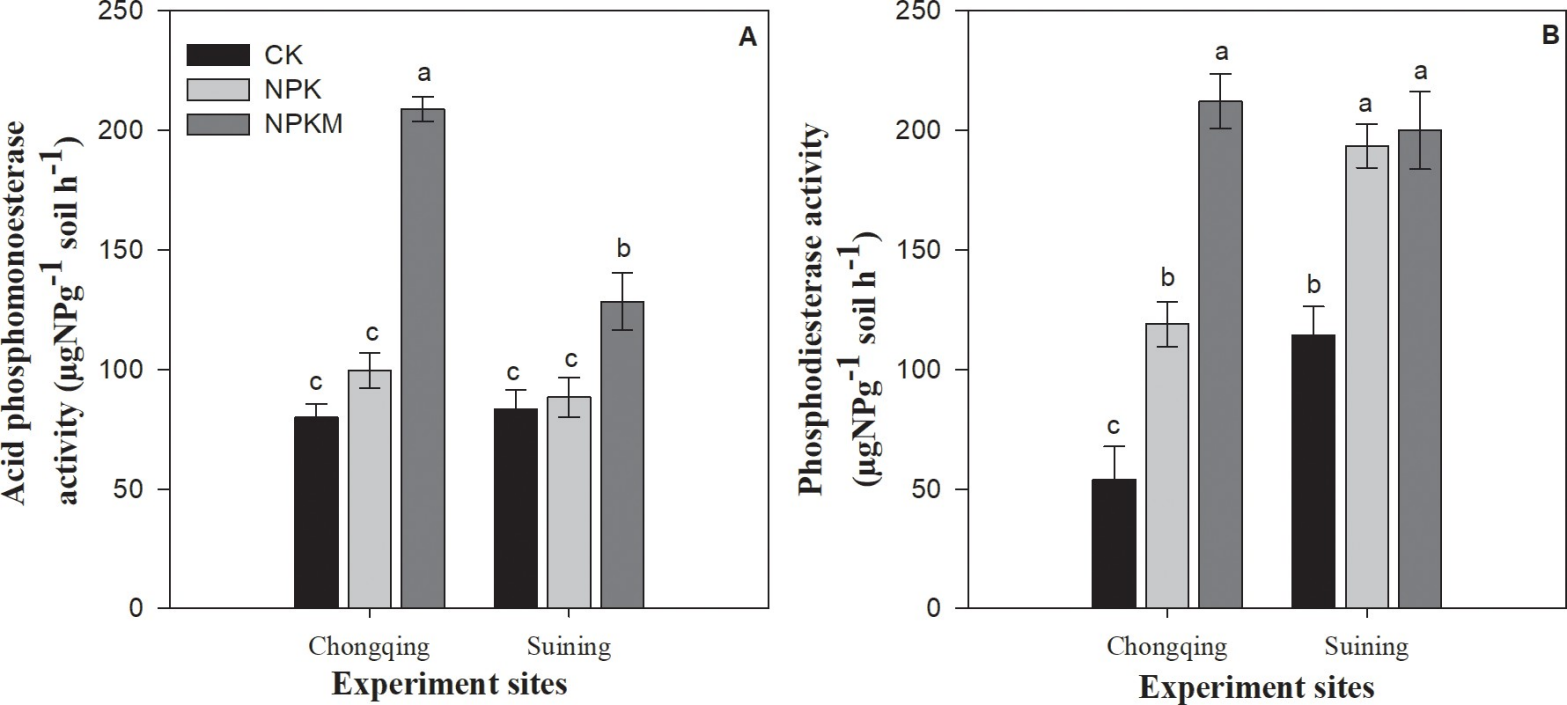
Effect of long-term fertilization on acid phosphomonoesterase (A) and phosphodiesterase (B) activities in paddy soils. Error bars represent ± standard deviations; different lower-case letters over the bars indicate significant (*P* ≤ 0.05) differences according to Tukey's HSD test.

Interactive effect of fertilization × site significantly (*P* ≤ 0.05) influenced acid phosphomonoesterase (AcP) and phosphodiesterase (PD) activities (Fig 3). The activities of AcP were not significantly different between the CK and NPK treatments at both sites. Compared with the CK treatment, the NPKM and NPK treatments increased the AcP by 161 and 24%, respectively at the Chongqing site and 54 and 6.4%, respectively in Suining. The increases in PD with NPKM and NPK treatments compared the CK treatment were 294 and 121% at Chongqing site and by 75 and 69%, respectively at the Suining site. Averaged across the treatments, the activity of AcP at Chongqing was by 23% greater than that at Suining, and the activity of PD at the Chongqing by 32% lower than that at the Suining site.

**Fig 4:**
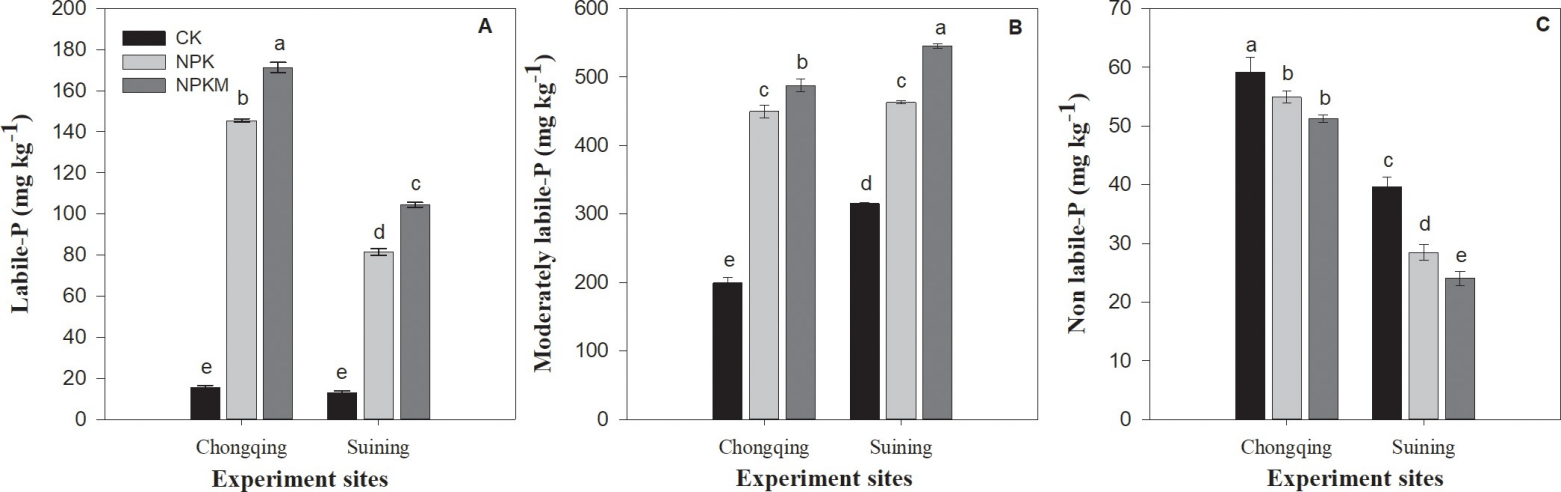
Effect of long-term fertilization on labile-P (A), moderately labile-P and non-labile-P pools in paddy soils. Error bars represent ± standard deviations; different lower-case letters over the bars indicate significant (*P* ≤ 0.05) differences according to Tukey's HSD test.

The soil P fraction distribution and uptake were significantly (*P* ≤ 0.05) influenced by the site × fertilizer interaction. Regardless of the treatments, all organic and inorganic fractions at the Chongqing were greater than those at the Suining site except NaOH-P_*i*_, HCl dil P_*i*_ and residual-P_*i*_ (Table 3). At the Chongqing site, compared with the CK treatment, the combined application of inorganic fertilizer and manure treatment increased NaHCO_3_-P_*i*_, NaHCO_3_-P_o_, NaOH-P_*i*_, NaOH-P_o_, HCl dil-P_*i*_, HCl conc P_*i*_ and HCl conc-P fractions by 1481, 368, 16706, 244, 106, 31 and 81% respectively and the NPK increased these factors by 1322, 197, 11798, 225, 96, 29.5 and 86%, respectively. At the Suining site, compared with the CK treatment, the NPKM treatment increased the NaHCO_3_-P_*i*_, NaHCO_3_-P_o_, NaOH-P_*i*_, NaOH-P_o_, HCl dil-P_*i*_, fractions by 888, 394, 5292, 77 and 37%, respectively and with the NPK treatment increased these fractions by 7.6, 1.4, 31, 0.3 and 0.28%. Residual-P was significantly decreased in the NPK and NPKM treatments compared with the CK treatment at both sites. In comparison with the NPK and CK treatments, long-term inorganic fertilizer plus manure application increased size of labile and moderately labile, while the non-labile pool was decreased in NPKM and NPK treatment compared with that in CK treatment (Fig 4).

**Table 3:** Concentrations of different fractions of phosphorus in the paddy soils affected by long-term fertilization at the two experimental sites.

Sites	Treatments	Labile-P (mg kg^-1^)		Moderately labile-P (mg kg^-1^)			Non-labile P (mg kg^-1^)		
		NaHCO3-Pi	NaHCO3-Po	NaOH-Pi	NaOH-Po	HCl. dil-Pi	HCl conc-Pi	HCl conc-Po	Residual-Pi
Chongqing	CK^1^	8.8 ± 1.1 c	7.0 ± 1.2 c	0.4 ± 0.1 c	12.4 ± 1.6 b	187 ± 6.5 b	25 ± 0.1 b	9 ± 0.1 b	25 ± 2.7 a
	NPK^2^	125 ± 1.1 b	20.8 ± 1.3 b	43 ± 0.8 b	40 ± 0.3 a	367 ± 9.8 a	32.6 ± 0.4 a	16 ± 0.2 a	6.0 ± 1.0 b
	NPKM^3^	139 ± 1.2 a	32.7 ± 1.6 a	60 ± 0.5 a	42.6 ± 1.3 a	384 ± 10 a	33 ± 2.8 a	16 ± 2.3 a	2.3 ± 0.6 b
Suining	CK	8.2 ± 0.5 c	4.8 ± 1.4 c	2.0 ± 0.2 c	23 ± 0.4 c	290 ± 1.9 c	14.5 ± 0.8 a	4.6 ± 0.9 a	20.6 ± 0.1 a
	NPK	70 ± 0.9 b	11.2 ± 1.1 b	64 ± 0.5 b	29.5 ± 0.3 b	370 ± 2.0 b	15 ± 0.1 a	2.9 ± 0.4 a	10.6 ± 1.1 b
	NPKM	81 ± 0.5 a	23.5 ± 1.1 a	108 ± 0.3 a	41 ± 1.8 a	396 ± 2.3 a	11.5 ± 0.9 b	3.4 ± 0.3 ab	9.2 ± 0.2 b
ANOVA	Site	***	***	***	ns	***	***	***	**
	Fertilization	***	***	***	***	***	***	***	***
	Site x Fertilization	***	***	***	***	***	***	***	***

Values are means ± standard deviations. For both soils, means followed by different letters are significantly (*P* ≤ 0.05) different from each other according to Tukey's HSD test. ns: non-significant

** Highly significant (*P* ≤ 0.01).

*** Highly, highly significant (*P* ≤ 0.001).

^1^ CK: unfertilized (control)

^2^ NPK: inorganic nitrogen, phosphorous and potassium

^3^ NPKM: inorganic NPK plus manure

**Fig 5:**
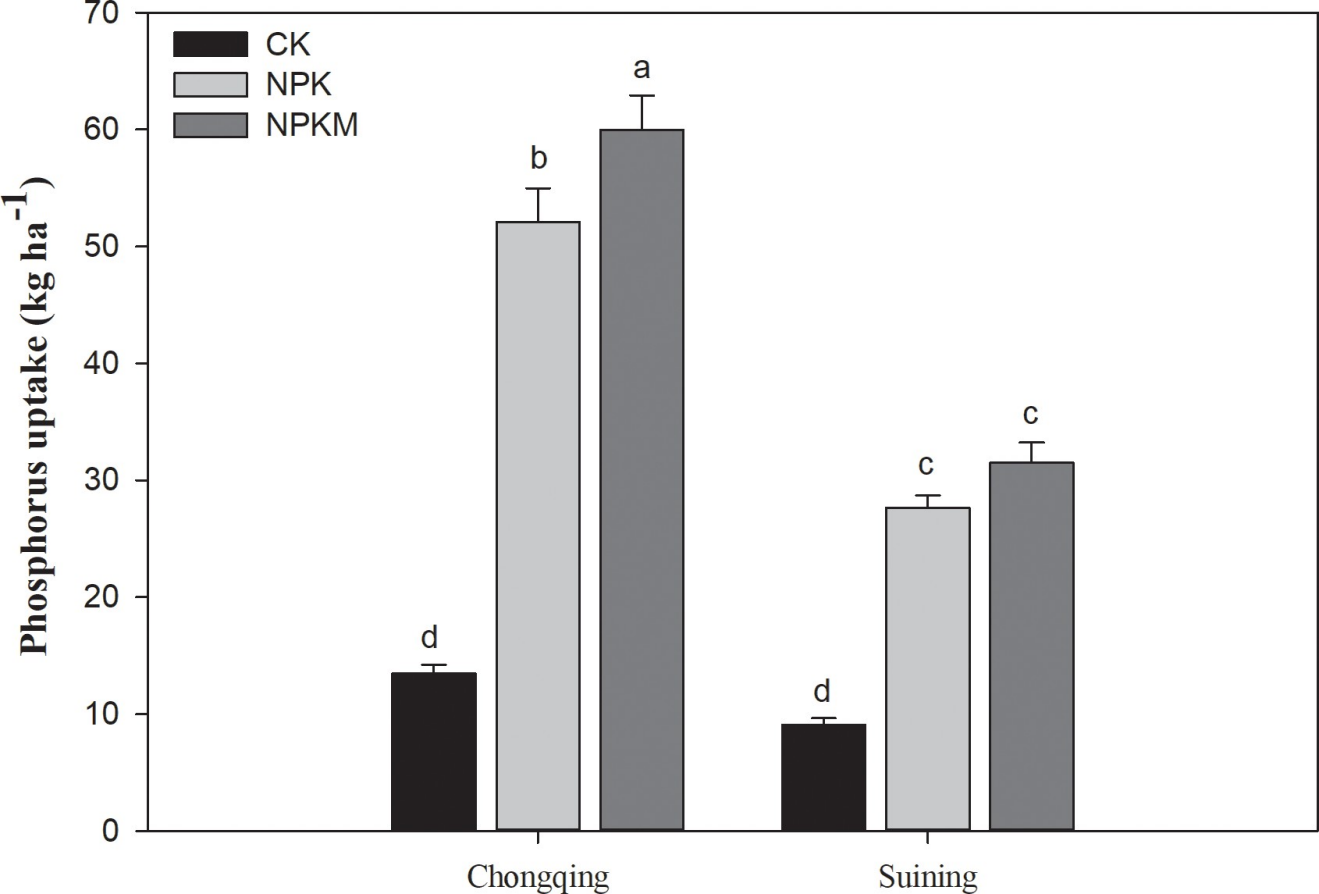
Effect of long-term fertilization on phosphorus uptake in rice crop in paddy soils. Error bars represent ± standard deviations; different lower-case letters over the bars indicate significant (*P* ≤ 0.05) differences according to Tukey's HSD test.

In comparison with the NPK and CK treatments, the long-term inorganic plus manure application treatment significantly (*P* ≤ 0.05) increased P uptake (Fig 5) by increasing P lability. Compared with the CK treatment, the NPKM and NPK treatments increased P uptake by 345 and 287%, respectively, at the Chongqing site and by 246 and 204%, respectively at the Suining site. Regardless of the treatments, the total P uptake at the Chongqing site was 46% greater than that at the Suining site.

### Relationship between soil C:N:P stoichiometry and phosphorus mobility

**Fig 6:**
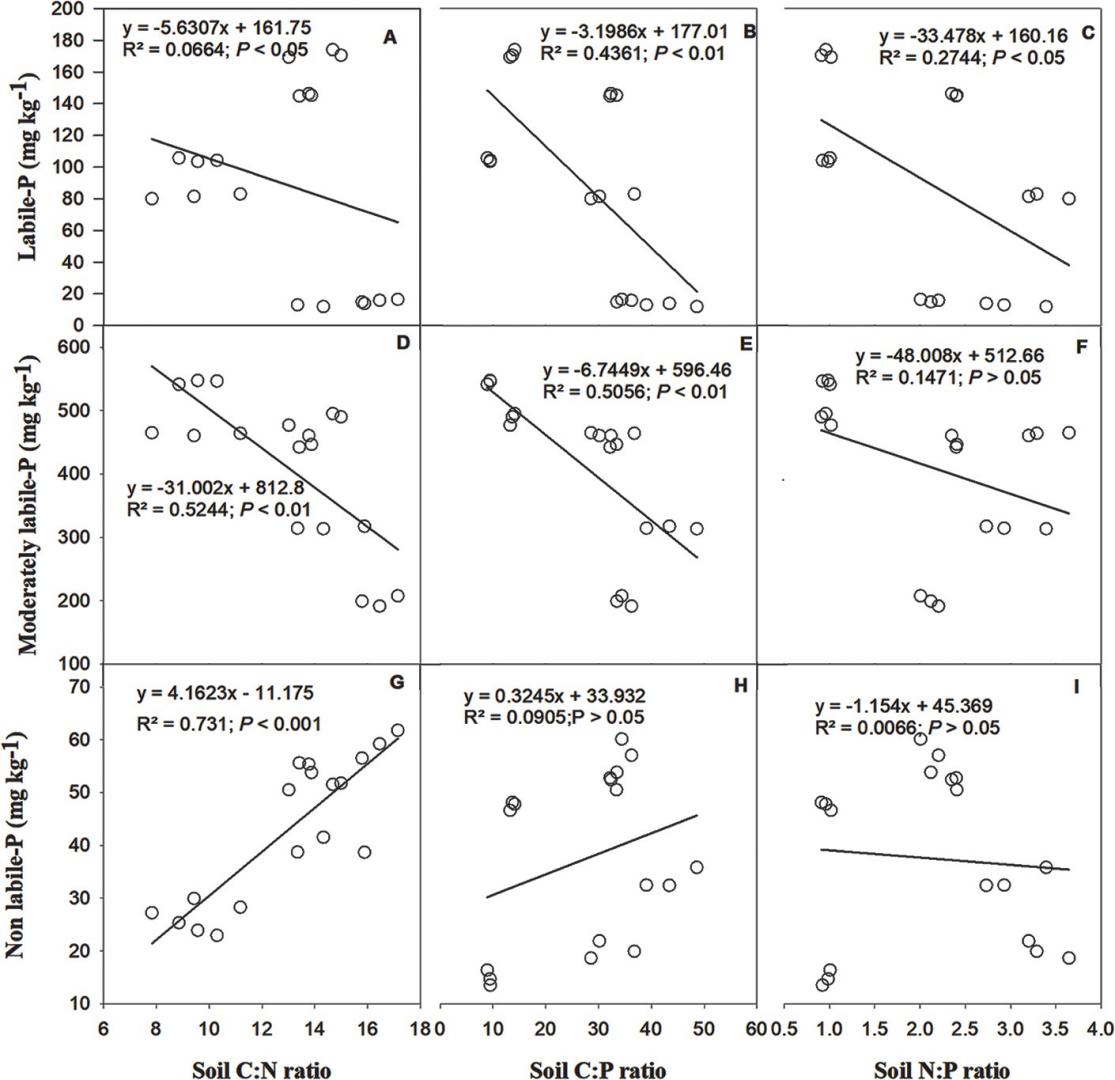
Linear regression indicating the relationship between soil C:N:P stoichiometry and phosphorus pools affected by long-term fertilization in paddy soil.

**Fig 7:**
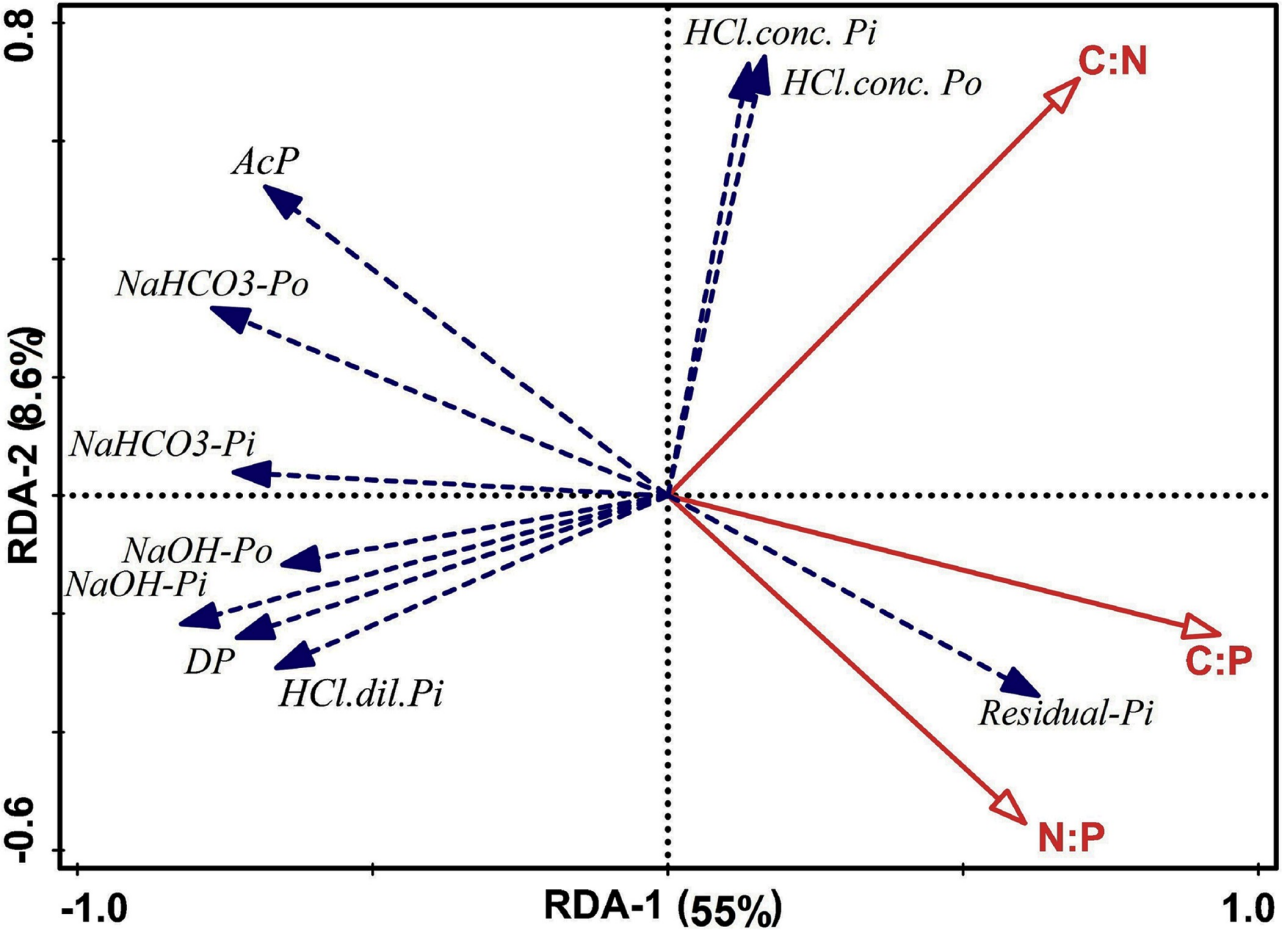
Redundancy analysis of the correlations between the soil C:N:P ratio, phosphorus fractions and phosphatase activities. Red lines indicate the effect of C:N, C:P and N:P ratios on phosphorus fractions and phosphatase enzyme activities. Blue dashed lines indicate the explanatory variables. Abbreviations: AcP: acid phosphomonoesterase activity; DP: phosphodiesterase activity.

The results from the linear regression showed that the soil C:N, C:P and N:P ratios were negatively correlated with labile-P and moderately labile-P pools (Fig 6). The C:N ratio had a highly significant (*P* ≤ 0.05) positive correlation (R^2^ = 0.73) with non-labile-P. The soil C:P and N:P ratios had a weak correlation with soil non-labile-P pool. Redundancy analysis (RDA) showed the effect of soil C:N:P stoichiometry on P fractions (Fig 7). Non-labile-P fractions especially residual-P, showed a close relationship with the N:P and C:P ratios, while, HCl conc-P_*i*_ and HCl conc-P_o_ showed a strong positive relation with the C:N ratio. RDA-1 explained 55% of the total variation, and RDA-2 explained 8.6% of the total variation.

**Fig 8:**
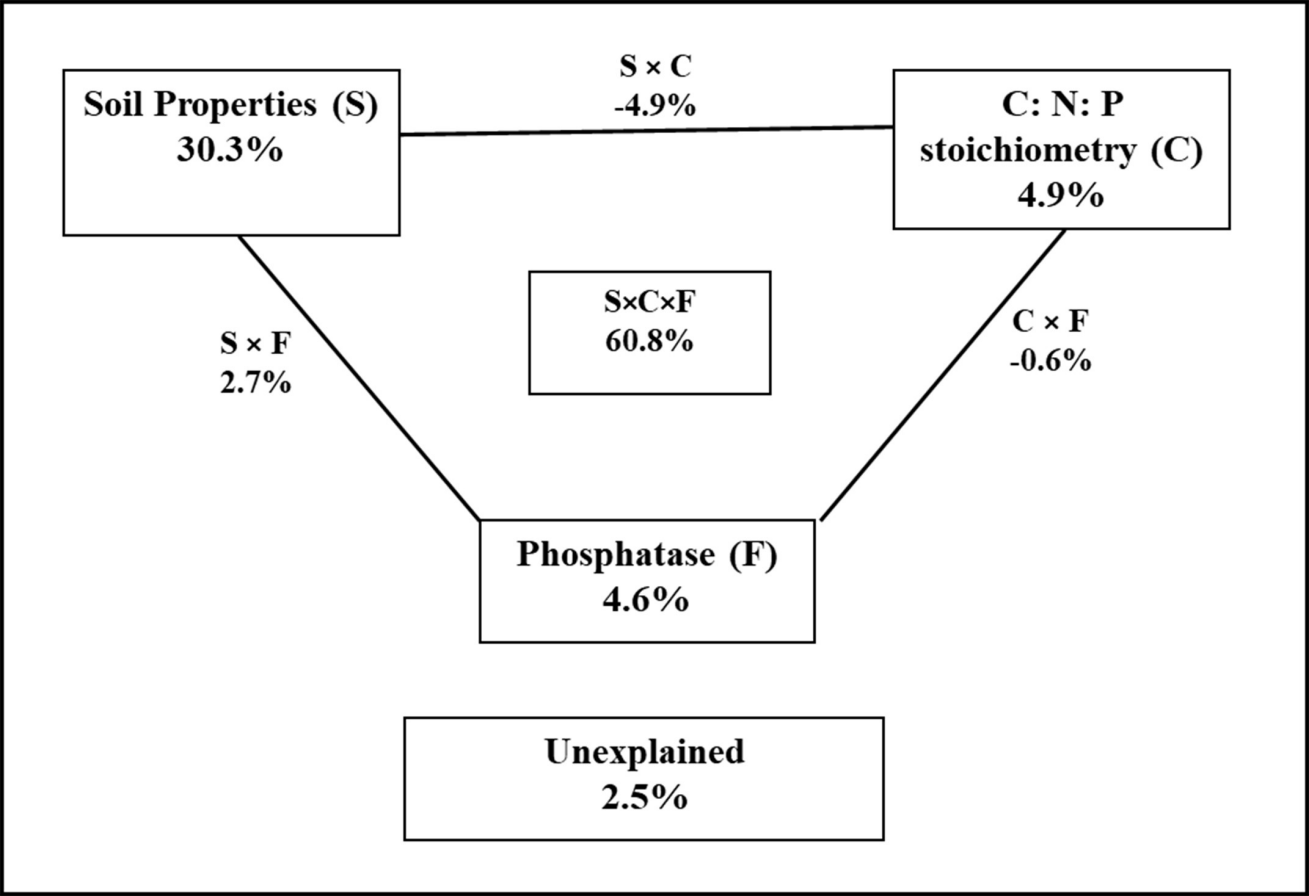
The variance partitioning analysis showing the proportional contribution (%) of soil properties (S), soil C:N:P stoichiometry (C), phosphatase (F) and their interactions on phosphorus fractions.

The variance portioning analysis (VPA) showed that soil properties, soil C:N:P stoichiometry and phosphatase totally explained 97.5% of the variation in the soil P fraction distribution (Fig 8). Soil properties included soil pH, SOM, total N, total P, and soil C:N:P stoichiometry (C:N, C:P and N:P ratios), and phosphatase activities included AcP and PD. Out of total variance the soil properties, soil C:N:P stoichiometry and phosphatase explained 30.3, 4.9 and 4.6% of the total variation, respectively, indicating the relative dominance of soil properties as a major factor.

**Fig 9:**
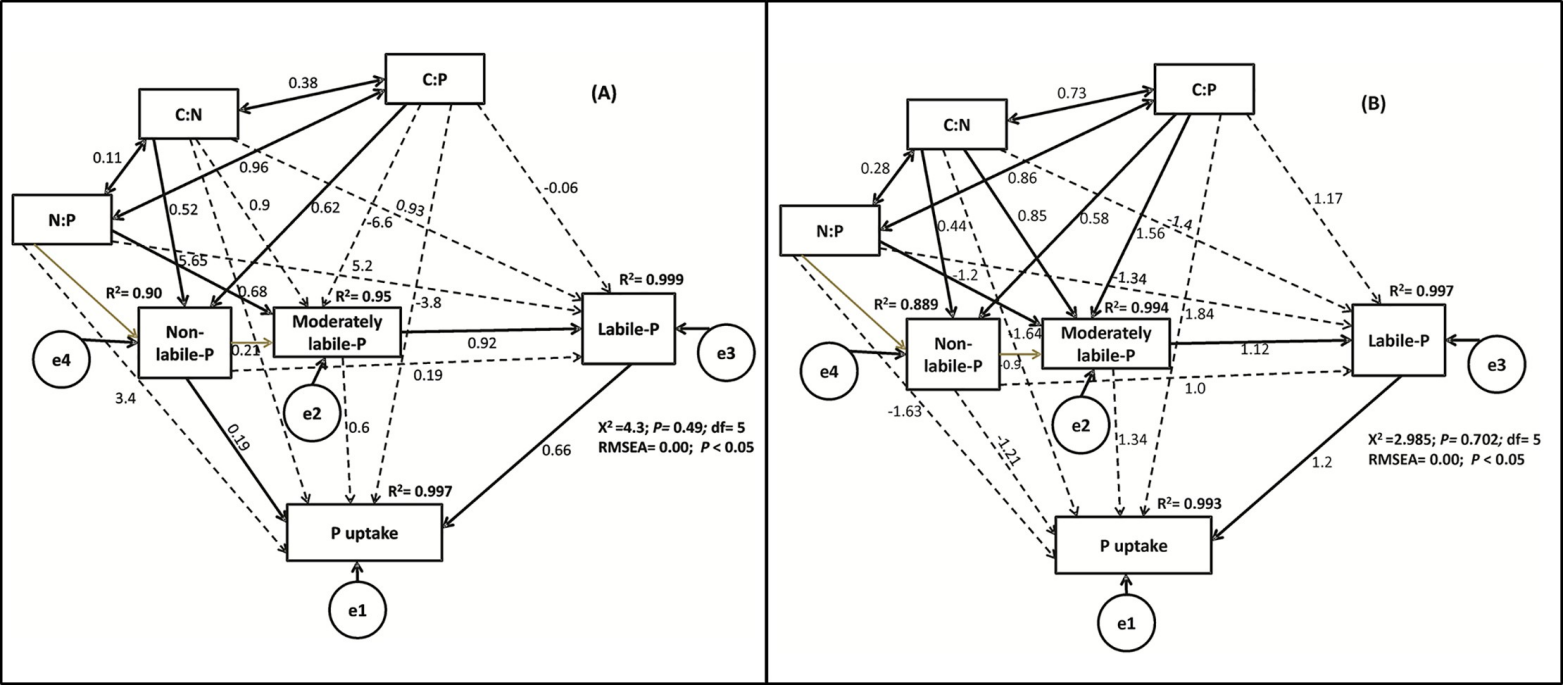
Structural equation modeling results for the direct and indirect effects of soil C:N:P stoichiometry on P lability and uptake at the Chongqing site (A) and the Suining (B) site. The numbers adjacent to the arrows are standardized path coefficients, which are analogous to relative regression weights, and indicative of the effect size of the relationship. The continuous and dashed arrows indicate direct and indirect relationships, respectively. Gray arrows indicate a non-significant effect. The proportion of explained variance (R^2^) appears above every response variable in the model. The goodness-of-fit statistics for each model are shown in the lower right corner. Chi/df = 0.86. RMSEA: Root mean square error of approximation.

We also investigated the direct, indirect and total effects of soil C:N:P stoichiometry on P pools and P uptake by a SEM pathway analysis (Fig 9A and 9B). The results showed that at the Chongqing site, SEM explained 99, 95, 90 and 99% of the variance in labile-P, moderately labile-P, non-labile-P and P uptake, respectively. N:P stoichiometry explained that the C:N and C:P ratios directly affected the non-labile P pool and indirectly affected the moderately labile and labile-P pools (Fig 9A). However, the C:N, C:P and N:P ratios indirectly affected P uptake by directly influencing P pools. Non-labile and labile-P both directly influenced P uptake, while moderately labile-P indirectly affected P uptake by directly affecting labile-P. At the Suining site, SEM explained 99, 99, 89 and 99% of the variance in labile-P, moderately labile-P, non-labile-P and P uptake, respectively. C:N, C:P and N:P ratios directly affected non-labile and moderately labile-P and indirectly affected labile-P (Fig 9B). However, P uptake was directly affected only by labile-P. Non-labile had no significant effect on moderately labile-P, but moderately labile-P had a direct effect on labile-P and an indirect effect on P uptake.

## Discussion

### Effect of long-term fertilization on soil properties, soil C:N:P stoichiometry and enzyme activities

Long-term inorganic and organic fertilizer application significantly influence soil properties [46]. In this study, we found a significant (*P* ≤ 0.05) effect of long-term chemical fertilizer and manure application on soil chemical properties (Fig 1) that affected soil C:N:P stoichiometry and enzyme activities. In this study, soil pH was significantly decreased by long-term chemical fertilization. Our results are consistent with those of Chen et al. [47] and Cui et al. [48], who investigated the long-term effects of organic and inorganic fertilization on soil acidification and found that the soils receiving different rates of chemical fertilizer inputs showed accumulation of exchangeable acidity and a reduction in soil pH. Soil pH may also influenced by the removal of base cations from soil with long-term cultivation [49]. Some studies have suggested that long-term manure application may increase the soil pH due to alkalinity of manure [48], a result is consistent with our results. In comparison with no fertilization and inorganic fertilization, long-term fertilization, especially combined application of NPK plus manure, highly increased SOM (Fig 1B). These results were also supported by the results of previous studies under long-term fertilization [49]. As a long-term combined application of manure and inorganic fertilizer enhances the productivity, an increase in carbon input in soil is also expected because it is a key driver of increased SOM [50,51]. The results were also consistent with findings of Ladha et al. [52], who found that long-term fertilization increases the soil organic matter, especially in paddy soil, due to a slower decomposition of SOM under flooded conditions [53]. However, due to different soil properties, the SOM content in both soils was significantly different, which could have been due to different soil properties and different N input rates (Table 2). SOM and nutrient availability are directly affected by fertilizer management [54]. In our study, the long-term combined application of inorganic fertilizer and manure also significantly increased the total and available N and P in at both experimental sites (Fig 1). The increases in nutrient availability with a continuous manure addition were observed in previous studies [46,55,56], and this increase could be due to the contribution of nutrient reserves in SOM-rich soils [47].

In comparison with no fertilization, long-term addition of chemical fertilization and manure in paddy soils changed the soil C:N:P stoichiometry (Fig 2). Surplus P accumulation in paddy soil under long-term combined inorganic fertilizer and manure application decreased the soil C:N, C:P and N:P ratio in the NPKM treatment in comparison with the inorganic fertilization and no fertilization treatments. This result was also due to the low supply of N and P in the CK and NPK treatments. Previous studies have also suggested that land-use changes such as cultivation practices and long-term fertilization may produce soil nutrient stoichiometric shifts, especially in paddy soils [57]. In our study, in both soils, the C:N and C:P ratios in the topsoil (0–20 cm) were approximately similar to the average ratios for China (12.3 and 52.7, respectively) [28] and to global average ratios [27]. The N:P ratio in our results was lower than those found by Wojciech et al. [58] in a forestland and by Wang et al. [57] in subtropical wetlands in China. In another study, an N:P ratio similar to our results was reported by Zhang et al. [59] during the afforestation on loess Plateau of China. Regardless of the treatments, the overall lower N:P may be due to the higher solubility of N than of P and the consequently higher losses of N than of P due to continuous flooding [57]. In addition, a lower N:P ratio under long-term combined application of inorganic fertilizer and manure supports the hypothesis of a more productive ecosystem with high P accumulation and a lower N:P ratio [60].

Enzyme activities play a vital role in the biochemical reactions of C, N and P cycling [61–63] and have different responses to different type of fertilization, soil and environment [64]. The changes in soil nutrient availability and stoichiometry under different types of long-term fertilization changed phosphatase activities (Fig 3). In both soils, the highest activities AcP and PD were found in combined inorganic and manure application treatment (Fig 3), which might be due to long-term manure addition and high C inputs compared with those in the NPK and CK treatments. Nayak et al. [65] also suggested that phosphatase activities increased by increasing soil organic matter. In another study Ohm et al. [66] also found positive relationship between soil carbon and phosphatase activities. Therefore, studying phosphatase activities could be helpful to understand the lability of P under long-term fertilization regulated by soil C:N:P stoichiometry.

### Phosphorus fractions, uptake and its relationship with soil C:N:P stoichiometry

Long-term fertilization significantly influenced P fractions distribution (Table 3) and P uptake (Fig 5). The changes in P fractions could be associated with soil properties. Many studies have explained the effect of soil pH, SOM, and P input rates on P fractions [67,68]. In our results, in contrast to previous studies, in comparison to no fertilization, long-term combined application of inorganic fertilizers and manure greatly increased the inorganic and organic fractions of P, except for the concentrated HCl organic and inorganic fractions of P (HCl conc-P_i_ and HCl conc-P_o_) and residual-P (Table 3). Yan [69] found that soil inorganic P fractions significantly increased following long-term inorganic fertilization but no corresponding differences were found in organic P fractions. In comparison to no fertilization, both the NPK and NPKM treatments decreased residual-P by increasing the labile-P. Because continuous manure application increased the lability of P by increasing both organic and inorganic fractions in labile pool which decrease the residual-P [20]. The P characteristics of organic material may also influence soil P content. For example, Li et al. [70] reported that in comparison to chemical fertilization, pig manure contained higher P_KCl_ content, therefore, the major available proportion of P in pig manure could be H_2_O-P or NaHCO_3_-P, which is readily available P for P uptake [70] Similarly, in our results, in comparison with inorganic and no fertilization, long-term combined application of inorganic fertilizer plus manure significantly increased P uptake in rice crops more by increasing the labile-P pool due to long-term manure addition (Fig 4). Our results are also consistent with results from previous studies [71]. Dobermann et al. [72] also found that P application with or without manure increased the soluble inorganic P fractions with little or no effect on residual-P. Different studies have reported that, soil containing high organic C content showed less P sorption and retention capacities for P [73], which indicates the high P availability for uptake.

Soil nutrient stoichiometry is more complex than in plants in the response to nutrient availability, because nutrient mobility in soil is influenced by both fertilization and plant uptake. In our results, soil C:N:P stoichiometry influenced P pools in paddy soils (Figs 6 and 7). Many studies have reported poor P fertility in flooded soil conditions [69]. However, there could be many soil and environmental factors that control the P mobility in soil [74,75], but most important factor controlling P mobility in paddy soil could be soil C:N:P stoichiometry. Because under long-term fertilization, the increase in carbon inputs by long-term combined inorganic fertilizer and manure addition shift the nutrient cycling in cropland, the decoupling of soil C:N:P in previous studies is well documented [76], however, the effect of soil C:N:P stoichiometry on P lability specially in paddy soil has not been studied before. The negative relationship of C:N and C:P ratios with labile-P and the positive relationship of C:N and C:P ratios with non-labile-P (Fig 6) indicate that long-term manure addition increased carbon input [51] and increased the non-labile fractions of P in paddy soils. The non-labile-P fractions contained organic P with different characteristics [69,74,77], which could be increased by long-term manure addition. Lehmann et al. [78] also reported that organic amendments mainly increase the organic fractions of P. In another study, González Jiménez et al. [77] also found that organic soils contained relatively higher concentrations of labile and non-labile organic proportion of P. Moreover, some studies have explained that manure addition in paddy soil decrease the decomposition rate of organic matter, especially under flooded conditions [53], which may affect the P release mechanism [20]. Therefore, in the present study high C:N and C:P ratios showed negative relationship with labile-P.

The SEM showed that soil C:N:P stoichiometry indirectly affected the P uptake by controlling P transformation and mobility in paddy soils (Fig 9). In a previous study, González Jiménez et al. [77] explained the pathways of P pools. However, to our knowledge no study before explained the soil C:N:P stoichiometric effect on P mobility in paddy soils by using pathway analysis. The C:N and C:P ratios directly affected the non-labile-P, possibly due to high carbon input under long-term manure addition. Soil organic matter and soil total P could be the main factors affecting P mobility in paddy soils [77]. Labile-P had a direct effect on P uptake (Fig 9A and 9B), because a labile-P pool is the sum of the all readily available P fractions, which have a direct effect on P uptake [79,80].The indirect effect of the soil N:P ratio on P uptake could be due to a positive direct effect of the N:P ratio on moderately labile-P. Previous studies have explained the synergistic effect of N on P uptake [23,57,81]. N application also affects P uptake by increasing biomass production and soil carbon inputs [82]. Interestingly, at the Chongqing site, non-labile-P had a direct effect on P uptake, and at the Suining site, non-labile-P had an indirect effect on P uptake (Fig 9B), because the soil P at the Chongqing site was relatively higher than that at the Suining site, which directly affected P uptake. These results are consistent with the results of previous studies, for example, in a path analysis study by González Jiménez et al. [77], soil residual-P directly affected the labile-P and therefore, increased the P uptake. In this study we found that the soil C:N:P stoichiometry controlled P lability in paddy soil. Therefore, our results emphasized the significance of soil C:N:P stoichiometric effect on P mobility and uptake in paddy soil. SEM explained the unique relationship between soil C:N:P stoichiometry and P mobility in paddy soils affected by long-term fertilization, and it could be helpful to understand the mechanism of P transformation in paddy soil affected by soil C, N and P cycle interaction. It also could be helpful to manage soil P fertility by understanding the mechanism of P mobility under long-term fertilization in paddy soils of the Chongqing and the Suining.

## Conclusion

We concluded that in comparison with no fertilization and chemical fertilization, long-term combined application of manure and chemical fertilizers significantly increased soil total and available nutrient content. In comparison to the CK and NPK treatments, the NPKM treatment significantly increased phosphatase activities in both soils. The soil C:N and C:P ratios was highest in the CK treatment, and no significant difference was observed between the NPK and NPKM treatments for the C:N ratio at both sites. While, the lowest soil C:P and N:P ratios were observed in the NPKM treatment at both sites. The NPKM treatment significantly increased P mobility and uptake, and reduced residual-P at both sites via increasing SOM. SEM pathways provided new insights into P lability mediated by the soil C:N, C:P and N:P ratios. The C:N, C:P and N:P ratios had a direct effect on moderately labile-P and non-labile-P, thus indirectly affecting mobile-P and P uptake. The soil organic matter and soil P content at the Chongqing site were relatively higher than those at the Suining site. Therefore, non-labile-P at the Chongqing site directly affected P uptake, and at the Suining site indirectly affected P uptake, indicating that soil C and P were two most important driving factors of P lability in paddy soil. Our findings suggest that soil nutrient stoichiometry is very important for managing the soil P fertility in paddy soil under long-term fertilization in these regions.
